# Monthly Summary

**Published:** 1893-02

**Authors:** 


### Monthly Summary.

THE "HERBST METHOD OF TREATING PULPS."—It may scarcely seem scientific to oppose any special line of practice which is apparently based on experimentation by mere arguments brought forward in the absence of experiments. And yet we cannot forbear taking issue in the main with the practice of Dr. Herbst as outlined in a paper read by Dr. C. F. W. Bodecker before the New York State Dental Society. Briefly stated, Dr. Herbst's treatment of pupils is as follows: Cobalt is applied and allowed to remain from two to three days. Then the coronal portion of the pulp is amputated with a large round bur in the engine, leaving the root-canals filled with pulp tissue. Over this pulp stump tin or gold foil is burnished by means of a rotary instrument in the engine. Then the remaining portion of the cavity is filled in the usual way.

We cannot see any reason for believing that cases thus treated will be successful. In the first place, cobalt will *kill* a pulp. It does not simply paralyze the coronal portion, but it destroys it to the apex of the root. It accomplishes this just as effectively as does arsenic employed in the usual form. If Dr. Herbst would wait one or two weeks after the application, instead of two or three days, he would find the pulp as dead as the proverbial "door-nail." For more than a year we have used nothing but cobalt in our practice for the destruction of pulps, and its action is precisely similar to arsenious acid, with the possible exception that its application does not ordinarily cause pain.

We all know, or ought to know, the result of leaving the canals filled with the dead pulp tissue and filling over it. The use of corrosive sublimate, as recommended, would probably tend to prevent sepsis for a time—in fact, the prolonged use of this agent will mummify a pulp stump quite effectively. But even a mummy is not safe in the root of a tooth. As soon as it absorbs moisture through the apex it is a mummy no longer, and soon becomes an abiding-place of putrefactive germs.

It is our conviction that the dentist who follows this practice extensively will reap a lamentable crop of failures, and the chief danger lies in the fact that it is advocated by reputable men who have sought to prove its propriety by experiments. It seems to us that time enough has not elapsed to render the experiments conclusive in such a line of practice as the treatment of pulps. A dead pulp is a treacherous enemy. It will often lie in wait a long time before manifesting its malignity.

We wish to warn the more confiding members of the profession against this new method. We believe it will prove on a line with some of the other fads of the past—in truth, it reminds us strongly of the craze which struck some very reputable members of the profession years ago when arsenic was hailed by them as the desideratum for the treatment of sensitive dentine. We will draw the mantle of charity over the scenes of those days in offices where this practice was followed.

We hope that those who are disposed to look with favor on this new treatment of pulps will "make haste slowly." Mistakes have been made before.—*C. N. J. in Review.*

---

WHAT IS IRON MADE OF?—This looks like a verg singular, not to say foolish question, and yet some chemists are beginning to doubt whether iron is really a chemical element. They think that instead of being an elementary substance it may be a highly complex compound, and that eventually means may be found of separating or isolating the bodies, or elements, of which iron is made up.

Different substances are ordinarily combined either by simple intermixture, as oxygen and nitrogen are intermixed in the air, or by solution, or by chemical combination.

But it has of late been suggested that there may be a fourth state of combination still more intimate than that which is implied by the usual expression, "chemical union." The combination of yet unrecognized elements which make what we call iron would be an example of this fourth state.

What this conception necessitates may be judged from the fact that it seems to do away with the atom as the smallest elementary particle of matter. In other words, it has been suggested that "atoms may be smashed." "Smashing" the atoms of iron would, according to this idea, be a method of discovering the elementary substances that compose it.—*Youth's Companion.*

---

A CHANCE FOR EVERY TOOTH.—Sir Andrew Clark, President of the Royal College of Physicians and Surgeons, is said to have told Mr. Gladstone that he had one mouth but thirty-two teeth, and that each mouthful of food should receive thirty-two bites in order to give every tooth a chance. A correspondent of *The Youth's Companion* writes that he was recently cognizant of an excellent proof of the truth of this statement. He says:

A sallow-faced, unhappy-looking man came to Doctor B——'s office one day when the writer chanced to be present. He wanted some medicine for dyspepsia.

Among other questions, the doctor asked, "How long a time do you usually spend at dinner?

"I dunno exactly," replied the patient: "ten or fifteen minutes, I guess."

"Does your food taste good?" Doctor B—— asked.

"That it does," was the reply, "but half an hour after I've eaten I'm near dying with distress."

"Do you drink much with your food—tea, coffee or water?"

"A pretty considerable amount," answered the man.

"Yours is a grave case," said the doctor, "but I can help you if you'll follow my directions."

Doctor B—— gave the man a dark-colored mixture in a bottle and said, Now, it is of the utmost importance that this medicine be taken properly. Put a teaspoonful into your cup of tea or coffee at each meal; stir it in thoroughly, and with each mouthful of food take a very small sip, and then chew, chew, chew, in order to mix it completely with the food. Do this and report to me in a week"

Two weeks later I saw this dyspeptic again, but I scarcely recognized him, he was so much improved in looks."

"That medicine of yours works like a charm," he said to the doctor. "I've about forgotten that I have a stomach."

"That is good," responded Doctor B——. "Continue taking it in the same way for three months, and you'll be a well man."

Then, as the man went out, Doctor B——said to me:

"The whole story of that man's cure is in the word *mastication*. It is merely what I said to him—chew, chew, chew. But he wouldn't have believed it without the medicine, which was the simplest. The man was bolting his food, and I stopped it. I've cured hundreds of dyspeptics in a similar way. Indeed, most dyspeptics might cure themselves if they would give every tooth a chance—thirty-two bites to a mouthful, with two for every tooth missing."

---

LYSOL.—Attention having been drawn by the recent cholera "scare" to the popularity of carbolic acid as a disinfectant, notice is being taken in medical circles of the even superior advantages for many purposes of the cresols as disinfectants. It was discovered that crude carbolic acid made soluble by the action of sulphuric acid surpassed in germicidal power an equally strong solution of pure phenol, besides which creolin, although free from carbolic acid, was proved to be of unmistakably superior disinfecting activity to the latter. Being insoluble in water, however, these cresols were neglected until the idea was hit upon of combining them with resin soap. Although very efficacious, these preparations were only emulsions; and it remained for the cresols to be made soluble, as now in the form of lysol, in order that what can be called the ideal soluble disinfectant should be made generally available. Lysol is produced by dissolving in fat, and subsequently saponifying, with the addition of alcohol, the fraction of tar oil which boils between 190° and 200° Cent. It is a brown, oily-looking, clear liquid, with a feabıy aromatic creosote-like odor. It contains 50 per cent of cr esols; and it is miscible with water to a clear, saponaceous, frothing fluid. It shows turbidity when mixed with hard water; but its disinfectant quality is not impaired thereby. It acts, to all intents and purposes, as a soap; and it is admirably adapted for use in surgical operations. According to German testimony, lysol is one of the most precious products of coal tar which chemistry has given to the service of mankind.—*Scientific American.*

